# Disability level and visibility: Associations with unmet academic accommodation needs and attitudes toward requesting accommodations

**DOI:** 10.1371/journal.pone.0342243

**Published:** 2026-02-09

**Authors:** Bryan R. Christ, Bani Malhotra, Olivia Chapman, Benjamin Ertman, Paul B. Perrin

**Affiliations:** 1 University of Virginia, Charlottesville, United States of America; 2 Virginia Commonwealth University, Richmond, United States of America; Lamar University, UNITED STATES OF AMERICA

## Abstract

**Purpose/objective:**

Despite legal mandates to provide appropriate accommodations for students with disabilities in the U.S., many report gaps between what they need and receive. This study examined the role of disability level, visibility, and demographic factors in predicting unmet academic accommodation needs and attitudes toward requesting accommodations.

**Research method/design:**

A sample of 409 adults who had had disabilities during their school years and who still had them completed an online survey assessing their current disability level, current disability visibility (invisible, semi-visible, or visible), unmet academic accommodation needs across all levels of schooling in aggregate, and current attitudes toward requesting academic accommodations.

**Results:**

Individuals with invisible disabilities in comparison to those with semi-visible or visible disabilities reported unmet needs for having a quiet or sensory room, extended time to take tests and exams, sensory objects (e.g., fidget toys), and an Individualized Education Plan. However, those with visible and semi-visible disabilities reported unmet need for having an educational assistant or tutor, recording equipment or a portable notetaking device, a modified or adapted course curriculum, and a computer, laptop or tablet with specialized software or apps. After controlling for disability severity and demographic variables, individuals with more visible disabilities had lower unmet academic accommodation needs compared to those with an invisible disability, as well as more positive attitudes toward requesting accommodations.

**Conclusion/implications:**

Assisting students with disabilities—especially those with invisible disabilities—may enhance disabled students’ experience of academic accommodations and empower them to advocate when those needs are unmet.

## Introduction

In 2023, 7.5 million students with disabilities in the U.S. received special education services, reflecting a 13% increase since 2013 [1]. These services are typically implemented through accommodations, or modifications in the setting, timing, presentation, scheduling, or response of school and curricular activities that allow students with disabilities to participate in the general education curriculum [2–4]. Despite the availability of special education services and relevant laws requiring the provision of these services for students with disabilities in K-12 and college education settings in the U.S., including the Individuals with Disabilities Education Act and Section 504 of the Rehabilitation Act of 1973, nearly 50% of students with disabilities who require accommodations report not receiving any, while 30% of those who do receive accommodations still feel they need additional support [2]. Understanding the disability-related and societal factors that impact unmet academic accommodation needs and self-advocacy for students with disabilities is crucial, as proper support can enhance academic performance [5] and foster students’ hope, efficacy, resilience, and optimism [6].

When examining why individuals with disabilities may not receive the accommodations they need, it is important to consider both individual and societal factors that may drive these unmet needs. To this end, this literature review considers individual factors associated with unmet academic accommodation needs established in the relevant literature and, where appropriate, connects them with societal factors or stigmas that may partly explain these relationships through the guiding lens of the social model of disability, which posits that individuals are disabled not by their disabilities but by the disabling barriers they face in society [7].

A variety of individual sociodemographic characteristics are associated with unmet academic accommodation needs and attitudes towards requesting accommodations among students with disabilities. Belonging to a racial/ethnic minority group [8], being a girl or woman [9,10], and disability diagnosis after the age of 11 [11] are associated with reduced likelihood of receiving appropriate academic accommodations. Regarding attitudes towards requesting accommodations, many students from racial/ethnic minority groups with disabilities hesitate to seek accommodations for fear of being perceived as doubly incompetent due to both their minoritized race/ethnicity and disability, which is in line with the social model of disability [12]. The stigma surrounding accommodation requests persists across genders, as girls fear appearing less competent and boys feel pressured to conform to competitive gender norms, again highlighting how the social environment structures experiences of disability [13]. This is particularly unfortunate because continued use of academic accommodations in college [5,14] and self-advocacy [15] are linked to higher grade point averages for students with disabilities which, as demonstrated in the non-disabled population, predict more favorable adult employment outcomes [16,17]. Findings on the relationship between rurality and academic accommodations are mixed, as rural schools tend to offer more inclusive education than urban schools, potentially reducing stigma, yet often lack resources adequately to support students with disabilities [18].

The type and level of a student’s disability may impact their specific unmet academic accommodation needs. Children with emotional or behavioral disabilities often do not receive the mental health services they need [19], and academic accommodations for students with invisible disabilities are often perceived as less appropriate [20]. Students with cerebral palsy who require technology-based supports (e.g., specialized software, recording or note-taking devices, audio/e-book devices) are more likely to have unmet academic accommodation needs compared to students with autism who may not require these supports [21], indicating that a lack of modern, technology-driven resources may disproportionately affect students with specific disabilities. Studies have shown that students with higher disability levels are more frequently provided support [10,22], highlighting a potential risk that students with milder disabilities may be less likely to receive needed accommodations.

In keeping with the social model of disability, many students with disabilities hesitate to advocate for academic accommodations due to the fear of being negatively perceived by their peers [23,24]. These fears are amplified for students with invisible disabilities who often feel the need to justify their accommodations [24] due to concerns that others may not believe they have a disability [25]. In contrast, students with visible disabilities can experience feelings of inadequacy, leading to academic overcompensation and potentially avoidance of using accommodations to prove their competence [25]. Although there is little research on how disability level affects attitudes towards accommodations, social acceptance in school is associated with the visibility—but not the severity—of the disability [26].

Taken together, this body of research reveals several important trends and disparities in how students with disabilities experience academic accommodations. Sociodemographic factors, disability type, and school context all influence whether students receive the support they need. However, much of this work has treated disability as a uniform category, overlooking the nuanced role of disability visibility—that is, how apparent a disability is to others—in shaping both unmet accommodation needs and students’ willingness to request support. While a few studies suggest that students with invisible disabilities face greater stigma and internalized pressure to justify their needs, there remains limited empirical research comparing unmet accommodation needs and accommodation-seeking attitudes across visibility levels, especially when controlling for disability severity. Little is known about how these patterns may persist into adulthood when individuals reflect back on their educational experiences. Addressing these gaps is critical to better supporting students whose needs may otherwise go unseen. As a result, the purpose of this study was to retrospectively examine how current sociodemographic factors, disability level, and disability visibility are associated with unmet academic accommodation needs across all levels of schooling in aggregate and current attitudes toward requesting accommodations in a sample of adults with disabilities.

## Methods

### Participants

The sample consisted of 409 participants aged 19–86 years old (*M* = 39, *SD* = 12.5) who all identified as having a disability or chronic health condition while going to school in the U.S. and passed quality control checks. They completed a 30-minute online survey with institutional review board approval from the host university. See Table 1 for sample demographics.

**Table 1:** Sample demographic characteristics.

Variable	Mean	*SD*
Disability Demographics Score (out of 6)	1.89	1.10
	***n***	**%**
Deaf or Difficulty Hearing	47	11.5
Blind or Serious Difficulty Seeing	42	10.3
Difficulty Concentrating, Remembering, or Making Decisions	279	68.2
Difficulty Walking or Climbing Stairs	117	28.6
Difficulty Dressing or Bathing	57	13.9
Difficulty Doing Errands Alone	232	56.7
Below Poverty Level	171	41.8
Employed (part or full time)	261	63.8
Gender		
Man	153	37.4
Woman	208	50.9
Transman	7	1.7
Transwoman	4	1
Gender non-binary/non-conforming	34	8.3
Other	3	0.7
Race/Ethnicity		
American-Indian/Native-American/Alaska-Native	4	1
Asian/Asian-American/Pacific Islander	13	3.2
Black/African-American	44	10.8
Latina/o/x or Hispanic	23	5.6
Multiracial/Multiethnic	24	5.9
White/European-American	300	73.3
Other	1	0.2
Education Level		
Grade school (elementary or middle)	5	1.2
High school/GED	49	12.0
Some college (no degree)	111	27.1
2-year/technical degree	54	13.2
4-year college degree	151	36.9
Master’s degree	35	8.6
Doctorate degree	4	1.0
Urbanicity		
Urban	131	32.0
Suburban	187	45.7
Rural	91	22.2
Number of Unmet Academic Accommodation Needs		
0	195	47.7
1	73	17.8
2	57	13.9
3	37	9
4	16	3.9
5	12	2.9
6	7	1.7
7	4	1
8	4	1
9	2	0.5
10	2	0.5

### Procedure

The survey consisted of two parts, delivered from October 18, 2023 – October 30, 2023, through Prolific, a data collection platform that handles participant recruitment and payment for academic researchers. Individuals (n = 970) first completed a one-minute initial screening survey on Prolific to determine eligibility for the study, as existing Prolific screening criteria do not include whether individuals had a disability or chronic health condition while going to school and lived in the U.S. during their years of schooling, which were our critical inclusion criteria. All participants had to be age 18 or older and were provided with an information sheet explaining the purpose of the study. Next, eligible individuals (n = 652) were invited to complete the full 30-minute survey, hosted on Qualtrics, on a first-come, first-served basis. We closed the survey after receiving 419 full responses, which was the maximum number of responses we were able to collect given the study’s budget. Participants received $.14 for completing the screening survey and $6 for completing the full survey, totaling $6.14 in compensation for full participation.

The full survey included several quality control procedures, including asking for participant age at both the beginning and end of the survey and ending the survey with an open-ended question (“In one sentence or less, what did you think was the purpose of this survey?”). We removed seven participants who did not pass both quality control checks and an additional three participants who reported that they did not have a disability or chronic health condition in the full survey despite indicating that they had one in the screening survey. This resulted in 409 final participants with complete survey data. The study was reviewed and approved as exempt by the University of Virginia Institutional Review Board (IRB) for the Social and Behavioral Sciences (Protocol #5640). In accordance with this approval and U.S. federal regulations for exempt, minimal-risk, anonymous survey research, participants provided informed consent by reviewing an IRB-approved information sheet and then affirmatively choosing to proceed with the survey. This procedure preserved participant anonymity while ensuring that all individuals were fully informed before participation. The consent process and study procedures complied with the ethical principles outlined in the Declaration of Helsinki.

### Measures

#### Demographics.

Participants reported their gender, age, race/ethnicity, education level, poverty status according to the U.S. federal poverty level, employment status, and urbanicity.

#### Disability.

We assessed current disability status using the Department of Health and Human Services Implementation Guidance on Data Collection Standards contained on the Centers for Disease Control and Prevention Disability Health Promotion website [27]. The survey contains six items that measure impairment caused by an individual’s disability or chronic health condition in the following areas: deaf or difficulty hearing; blind or serious difficulty seeing; difficulty concentrating, remembering, or making decisions; difficulty walking or climbing stairs; difficulty dressing or bathing; and difficulty doing errands alone such as visiting a doctor’s office or shopping. Participants indicate impairment in each domain on a binary (yes/no) scale, and in this study the items were summed for a total score out of 6. The survey also included a question that measures disability visibility (“How visible to other people is your disability or chronic health condition that causes you the most daily difficulty?”) with three response options (visible, semi-visible, invisible), which we used to assess disability visibility in this study. We also assessed current disability level using a modified version of the Pain Disability Index (PDI) [28], which measures the level of impairment caused by an individual’s disability or chronic health condition in seven areas: family/home responsibilities, recreation, social activity, occupation, sexual behavior, self-care, and life-support activities. Participants respond on a 10-point Likert scale. The seven items are summed to get an overall score with higher scores indicating higher levels of disability-related impairment.

#### Accommodation attitudes/experiences and accommodations received.

We measured attitudes toward and experiences with requested and received accommodations through two primary measures: the Attitudes Towards Requesting Accommodations (ATRA) scale [29] and Education Accommodation (EDU_Q65 and EDU_Q70) items from the 2022 version of the Canadian Survey on Disability [30]. ATRA is a 32-item scale answered using five-point Likert responses that measures an individual’s attitude toward requesting accommodations in four main areas (academic integrity, disability disclosure, disability acceptance, and the accommodations process). Items are summed to create a total score, with higher scores reflecting more positive attitudes towards requesting accommodations. Education Accommodation questions from the Canadian Survey on Disability ask individuals to separately indicate the education accommodations that they needed and received from an exhaustive list. We asked participants to respond to these questions considering their educational experiences as a whole, meaning considering accommodations that they felt they had needed and that they had received throughout their educational experiences without reference to a specific level of schooling. Traditionally, scores are calculated as a percentage of total met accommodation need by dividing the sum of received accommodations by the sum of needed accommodations. However, some individuals reported receiving accommodations that they did not need, which created a division by 0 error. As a result, we calculated the total score by subtracting the sum of received accommodations from the sum of needed accommodations such that higher scores indicate greater unmet accommodation need. Individuals who received accommodations they did not need had negative scores, and we reassigned these values to zero to indicate they had no unmet accommodation need.

### Data analysis

We conducted all analyses using Python 3.11.5. We used the Pandas and NumPy packages to calculate descriptive statistics, bivariate correlations, and Cronbach’s α, and the statsmodels package for implementing ordinal regressions. We included descriptive statistics and bivariate correlations to show which specific unmet academic accommodation needs were prevalent in our sample and to explore relationships among variables before adding controls into our regression analyses, respectively. We included Cronbach’s α to demonstrate the statistical reliability of the survey instruments used in the study. We used ordinal regressions because they are designed to model ordinal outcomes, or ones that have integer-only, numeric, and ordered categories, and both unmet academic accommodation needs and ATRA scores are naturally ordinal because they are ordered and only take integer values. Ordinal regressions model the log odds of increasing from one integer value of the outcome to another, so coefficients for predictors in these regressions represent the log odds of increasing to a higher level of the outcome variable with a one unit increase in the predictor. Coefficients that are positive represent higher odds of increasing to a higher level of the outcome variable, whereas negative coefficients represent lower odds of increasing to a higher level of the outcome variable. Using the logistic function, it is also possible to convert coefficients to probabilities of moving to a higher level of the outcome variable, which can be easier to interpret. When considering coefficients as probabilities, a probability < 0.5 indicates higher levels of the predictor decrease the likelihood of having a higher level of the outcome variable, a probability > 0.5 indicates higher levels of the predictor increase the likelihood of having a higher level of the outcome variable, and a probability = 0.5 indicates the predictor has no effect on the outcome variable.

## Results

### Descriptive statistics and reliability metrics

Table 2 displays specific unmet academic accommodation needs by disability visibility. As shown in the table, when considering academic accommodations where 10% or more of the subsample had unmet need, individuals with invisible disabilities in comparison to those with semi-visible or visible disabilities reported unmet needs for having a quiet or sensory room, extended time to take tests and exams, sensory objects (e.g., fidget toys), and an Individualized Education Plan. However, those with visible and semi-visible disabilities reported unmet need for other types of academic accommodations where 10% or more of the subsample had unmet need, including having an educational assistant or tutor, recording equipment or a portable notetaking device, a modified or adapted course curriculum, and a computer, laptop or tablet with specialized software or apps.

**Table 2:** Unmet academic accommodation need by disability visibility.

	Invisible Disability		Semi-visible Disability		Visible Disability	
Unmet Academic Accommodation Need	n	%	n	%	n	%
Quiet room or sensory room	**57**	**22.0**	24	20.5	4	12.1
Extended time to take tests and exams	**48**	**18.5**	14	12.0	5	15.2
Sensory objects (e.g., fidget toys)	**39**	**15.1**	15	12.8	2	6.1
Educational assistant or tutor	30	11.6	**19**	**16.2**	4	12.1
Recording equipment or a portable note-taking device	26	10.0	15	12.8	**8**	**24.2**
Modified or adapted course curriculum	27	10.4	13	11.1	**4**	**12.1**
Individualized Education Plan (e.g., IEP, PLP)	**28**	**10.8**	9	7.7	2	6.1
Computer, laptop or tablet with specialized software or apps or other adaptations to help with your condition	23	8.9	5	4.3	**4**	**12.1**
Cell phone, smartphone or smartwatch with specialized features or apps to help with your condition	19	7.3	9	7.7	**3**	**9.1**
Device for playing audio books or e-books	15	5.8	7	6.0	**3**	**9.1**
Textbooks in e-format	15	5.8	7	6.0	**2**	**6.1**
Large print reading materials	6	2.3	**5**	**4.3**	1	3.0
Special education classes	**8**	**3.1**	2	1.7	1	3.0
Screen magnification software	5	1.9	3	2.6	**2**	**6.1**
Magnifiers	5	1.9	1	0.9	**2**	**6.1**
Attendant care services	2	0.8	3	2.6	**2**	**6.1**
Speech therapist	4	1.5	2	1.7	**1**	**3.0**
Closed-circuit devices (e.g., CCTV)	1	0.4	**1**	**0.9**	0	0.0
Braille Refreshable Display device, braille note taker, braille reading materials or a manual brailler	0	0.0	0	0.0	0	0.0
Sign language interpreter	0	0.0	0	0.0	0	0.0

*Note. Bold denotes the group with the highest unmet need for each type of academic accommodation.

Table 3 presents bivariate correlations among all primary study variables, including unmet academic accommodation need, attitudes towards requesting accommodations (ATRA), disability demographics score, disability severity, and disability visibility. Most correlations were significant at the *p* < .05 level except for between disability visibility and unmet academic accommodation need, disability demographics score and ATRA, and disability severity and ATRA. Using the well-established cutoffs for small (*r*≥ .10), medium (*r*≥ .30), and large (r ≥ .50) correlations from Cohen [31,32], small but significant correlations occurred between disability demographics score and unmet academic accommodation need (*r* = 0.23), disability visibility and ATRA (*r* = 0.12), and disability visibility and disability severity (*r* = 0.20). Medium and significant correlations occurred between disability severity and unmet academic accommodation need (*r* = 0.32), disability severity and disability demographics score (*r* = 0.46), and disability visibility and disability demographics score (*r* = 0.32). Table 4 presents Cronbach’s α for the three scales used in this study. As shown in the table, all three scales meet the threshold for acceptable reliability, with the ATRA and Disability Severity scales meeting the threshold for good reliability.

**Table 3:** Bivariate correlation matrix among primary study variables.

	1	2	3	4
1. Unmet Academic Accommodation Need				
2. ATRA	−0.08			
3. Disability Demographics Score	0.23*	0.06		
4. Disability Severity (PDI)	0.32*	−0.02	0.46*	
5. Disability Visibility	0.01	0.12*	0.32*	0.2*

*Note.* * = *p* < 0.05.

**Table 4:** Cronbach’s α for primary study scales.

	Cronbach’s α
Unmet Academic Accommodation Need	0.74
ATRA	0.83
Disability Severity (PDI)	0.80

### Ordinal regression models

#### Unmet academic accommodation need.

Table 5 shows results for the three ordinal regression models with increasing steps to predict unmet academic accommodation needs using disability visibility. The first step included only disability visibility and displayed a small negative and statistically non-significant relationship between disability visibility and unmet academic accommodation needs. The second step controlled for two measures of disability level to identify whether individuals with more invisible disabilities had higher unmet academic accommodation need when controlling for disability severity. The second step showed a large negative and significant relationship between disability visibility and unmet academic accommodation need, meaning that those with invisible disabilities were more likely to have higher unmet academic accommodation needs when controlling for disability level. The coefficients for disability demographics score and disability level were smaller in magnitude, positive, and statistically significant, suggesting that those with higher disability levels generally had higher unmet academic accommodation need.

**Table 5:** Ordinal regressions for unmet academic accommodation need.

	(1) Visibility Only		(2) Visibility and Disability Level		(3) Visibility, Disability Level, and Demographics	
	Coefficient	Probability of Moving Up	Coefficient	Probability of Moving Up	Coefficient	Probability of Moving Up
Disability Visibility	-0.09 (0.15)	0.48	-0.46** (0.16)	0.39	-0.37* (0.16)	0.41
Disability Demographics Score			0.25* (0.10)	0.56	0.29** (0.10)	0.57
Disability Level			0.04** (0.01)	0.51	0.04** (0.01)	0.51
Below Poverty Level					0.13 (0.24)	0.53
Age					0.00 (0.01)	0.50
Employed					0.16 (0.24)	0.54
Female or Trans					0.13 (0.20)	0.53
College Degree					-0.24 (0.22)	0.44
Rural					-0.14 (0.23)	0.47
Underrepresented Minority					-0.03 (0.23)	0.49

*Note:* Ordinal regression coefficients and corresponding probabilities for three fitted models for unmet academic accommodation need: 1) disability visibility only, 2) disability visibility and disability level, and 3) disability visibility, disability level, and demographics. Probabilities represent the probability of having an additional unmet academic accommodation need with a one unit increase in the independent variable. Probabilities < 0.5 indicate decreased likelihood of higher unmet academic accommodation need, probabilities > 0.5 indicate increased likelihood of higher unmet academic accommodation need, and probabilities = 0.5 indicate the independent variable has no effect on unmet academic accommodation need. Standard errors are in parentheses and a * or ** denotes a coefficient is significant at the *p*< 0.05 or *p*< .01 level, respectively.

In the third step, we added in controls for participant demographics to see whether these variables are responsible for the relationship we found between disability visibility and unmet academic accommodation need in step two. When adding in controls for participant demographics, the coefficient for disability visibility became smaller in magnitude but stayed statistically significant, suggesting that demographics may explain part but not all of the relationship between disability visibility and unmet academic accommodation need. With these demographic controls, the coefficients for the measures of disability level were similar in magnitude to the second step and still statistically significant, suggesting demographics do not mediate the relationship between disability level and unmet academic accommodation needs. None of the demographic variables were significant predictors of unmet academic accommodation need.

### Attitudes towards requesting accommodations

To explore why individuals with invisible disabilities might have higher unmet academic accommodation needs when controlling for disability level, we ran another multi-step ordinal regression model to predict ATRA scores using the same approach as above. As shown in Table 6, in all three steps, the coefficient for disability visibility was relatively large, positive, and statistically significant. This suggests that even when controlling for disability level and demographics, those with more visible disabilities had more positive attitudes toward requesting academic accommodations, which may partly explain why they had lower unmet academic accommodation need when controlling for disability level. The coefficients for the measures of disability level were small and not significant, suggesting that disability level did not impact participants’ attitudes towards requesting accommodations. None of the demographic variables were significant predictors of ATRA scores.

**Table 6:** Ordinal regressions for attitudes towards requesting accommodations.

	(1) Visibility Only		(2) Visibility and Disability Level		(3) Visibility, Disability Level, and Demographics	
	Coefficient	Probability of Moving Up	Coefficient	Probability of Moving Up	Coefficient	Probability of Moving Up
Disability Visibility	0.33* (0.14)	0.58	0.34* (0.14)	0.58	0.35* (0.15)	0.59
Disability Demographics Score			0.05 (0.09)	0.51	0.03 (0.09)	0.51
Disability Level			−0.01 (0.01)	0.5	−0.01 (0.01)	0.5
Below Poverty Level					−0.25 (0.21)	0.44
Age					0.00 (0.01)	0.5
Employed					−0.39 (0.21)	0.4
Female or Trans					0.13 (0.18)	0.53
College Degree					0.17 (0.20)	0.54
Rural					−0.25 (0.22)	0.44
Underrepresented Minority					0.41 (0.21)	0.6

*Note:* Ordinal regression coefficients and corresponding probabilities for three fitted models for Attitudes Towards Requesting Accommodations (ATRA): 1) disability visibility only, 2) disability visibility and disability level, and 3) disability visibility, disability level, and demographics. Probabilities represent the probability of having a one unit higher ATRA score with a one unit increase in the independent variable. Probabilities < 0.5 indicate decreased likelihood of higher ATRA scores, probabilities > 0.5 indicate increased likelihood of higher ATRA scores, and probabilities = 0.5 indicate the independent variable has no effect on ATRA scores. Standard errors are in parentheses and a * or ** denotes a coefficient is significant at the *p* < 0.05 or *p* < 0.01 level, respectively.

## Discussion

This study examined the potential impact of disability level and disability visibility on unmet academic accommodation needs and attitudes towards requesting accommodations for individuals with disabilities. Building on previous research [2], about half of the sample reported unmet academic accommodation needs, with varying specific needs among those with visible, invisible, and semi-visible disabilities. Bivariate correlations between the variables demonstrated that disability level had a positive, medium-sized correlation with unmet academic accommodation need and disability demographics score, while disability visibility had a positive, medium-sized correlation with disability demographics score. When controlling for disability level, individuals with more visible disabilities had a lower probability of having unmet academic accommodation needs, consistent with prior research on barriers and difficulties of receiving academic accommodations for students with hidden or invisible disabilities [20,33–35]. Individuals with more visible disabilities had more positive attitudes toward requesting academic accommodations. Disability level did not predict attitudes towards requesting accommodations, consistent with previous findings [26]. No significant sociodemographic predictors of unmet academic accommodation needs or attitudes towards requesting accommodations were identified. These findings demonstrate that participants with invisible disabilities are more likely to have higher unmet academic accommodations and are less likely to request those accommodations, drawing attention to factors that may impact their educational experience.

Notably, individuals with invisible disabilities reported the highest unmet accommodation needs for quiet or sensory room, extended time to take tests and exams, sensory objects (e.g., fidget toys), and an Individualized Education Plan. Those with visible and semi-visible disabilities reported the most unmet needs for an educational assistant or tutor, recording equipment or a portable notetaking device, a modified or adapted course curriculum, and a computer, laptop or tablet with specialized software or apps. These align with the reported accommodations and supports provided and generally recommended for students at the K-12, secondary, and post-secondary levels [36,37]. Previous research has highlighted the academic benefits of specific accommodations like the importance of small group instruction and tutoring [38,39], extended time to demonstrate knowledge [3], a quiet room or environmental supports for sensory regulation strategies [40,41], adjusted or adapted education plans [42], and the use of specific software and note taking devices [43]. Thus, not providing students with disabilities with the academic accommodations they need could lead to adverse educational outcomes.

Bivariate correlations revealed medium and significant positive correlations between disability severity and unmet academic accommodation need, disability severity and disability demographics score, and disability visibility and disability demographics score. It is likely that individuals with more severe disabilities had higher unmet accommodation need simply because they require more accommodations, although this relationship has not been significantly explored in the relevant literature. The correlation between disability severity and disability demographics score is indicative of the fact that one measure of disability severity will naturally be highly correlated with another, and the correlation between disability visibility and disability demographics score is due to the fact that both measures predominately assess physical manifestations of disability.

Participants with invisible disabilities had higher unmet academic accommodation needs and more negative attitudes towards requesting accommodations even when controlling for disability level and demographics. It is possible that participants with more visible disabilities have a lower likelihood of having unmet needs due to their positive attitude towards seeking accommodations. Disability type has also been found to be a factor influencing accommodation request and fulfilment [20]. Even though there may be legal mandates for granting accommodations in accordance with the Individuals with Disabilities Education Act (IDEA) and Section 504 of the Rehabilitation Act of 1973, those with invisible disabilities experience frequent barriers that hinder their access to and perception of accommodations. Some of these barriers include lacking self-awareness regarding legal rights or self-advocacy skills, hesitancy in disclosing their disabilities fearing stigma from disclosure, renouncing disability identity to avoid labelling, lack of awareness of available supports, and inhibition in requesting accommodations and supports [35,44,45]. Relatedly, is also possible that individuals with more invisible disabilities have higher unmet need due to them having more negative views towards seeking accommodations, which could partly be driven by societally-instilled stigmas surrounding pressures for individuals with invisible disabilities to “pass” as normal or else have to “prove” their disability [46,47]. Societal and environmental barriers, including attitudinal, architectural, administrative, and programmatic factors have also been identified as impacting accommodation experiences [48]. Taken together and in keeping with the social model of disability, our findings and those from the related literature show that there is a complex interplay between individual characteristics and societal norms around and barriers to accommodations that may explain why individuals with more invisible disabilities have higher unmet academic accommodation need and more negative attitudes towards requesting these accommodations.

Also in keeping with the social model of disability, faculty or teacher knowledge, attitude, and willingness greatly shape disabled students’ success in acquiring or requesting accommodations [34]. Uninformed or untrained faculty or teachers harboring negative perceptions, skepticism, and reluctance to accommodate, particularly for invisible disabilities, worsens these students’ experiences [49–51]. Furthermore, there may be gaps in policy implementation and available resources. Barriers may exist in terms of teacher or faculty workload, lack of training in inclusive practices, and insufficient funding for assistive tools, coordinated services, and individual support [48]. Challenges in appropriate assessment and documentation of disability for securing accommodations have also been highlighted as a potential barrier, especially for those who must prove their need for support [25,52]. In these ways and as posited in the social model of disability, the broader school context may be another disabling factor shaping the higher unmet academic accommodation needs and more negative attitudes towards requesting accommodations for those with invisible disabilities in addition to the individual characteristics and societal norms discussed above.

Both measures of disability severity were statistically significant predictors of increased likelihood of unmet academic accommodation need, which is consistent with the bivariate correlation results. Academic accommodation experiences may also vary depending on the disability category, leading to unmet needs. While institutions may improve architectural accessibility (ramps, doors, elevators) for students with visible disabilities, other environmental barriers may persist. For instance, students with chronic illness often receive insufficient support for their academic and social-emotional needs, including high absenteeism [53]. Additionally, children with learning disabilities, ADHD, autism spectrum disorder, and those with emotional-behavioral problems often do not receive the services they need [19] or may pose greater challenges for teachers compared to those with sensory/motor disabilities [48]. Our study found that demographics did not significantly mediate the relationship between either disability level or visibility and unmet academic accommodation needs, or between disability visibility and attitude towards requesting accommodations. Future research should systematically examine the role of gender, race/ethnicity, and socioeconomic or funding status of the school/area to fully ascertain their impact on unmet accommodation needs and attitude towards seeking these accommodations among various stakeholders [9,18,54,55].

### Implications and future directions

This study provides important insight on unmet academic accommodation needs, disability visibility, and perceptions towards requesting accommodations. Given that students with invisible disabilities may face difficulties in having needs met or requested, future research is needed to understand the role of specific disability type, for instance hidden disability type such as learning, cognitive, or psychiatric conditions, and associated challenges or stigma towards accommodations [56]. It is important to examine the personal, social, and societal factors that limit the academic accommodations students receive and deter them from requesting them. A comprehensive student-centered approach to identify needs and potential accommodations at various stages of schooling based on disability visibility may be required. This study provides important insight into types of unmet needs which may have a direct impact on students’ academic experience, with implications for clinicians and educators that recommend accommodations. Based on participants’ responses, educators can especially consider providing a quiet room, sensory tools, time extensions, and individualized education plans for students with invisible disabilities and small group instruction, tutoring, recording devices and specialized software or tools to those with semi-visible and visible disabilities.

The relationship between disability visibility and attitude towards requesting accommodations is particularly important because students with invisible disabilities must be able to advocate for themselves to succeed in procuring accommodations that are functional and effective, particularly as they get older. Since peer and faculty/teacher perceptions of disabilities and accommodations can be critical, educators and institutions can consider implementing proactive supports through awareness and comprehensive faculty and teacher development programming that encourage inclusion and accommodations with discretion, rather than those based on interrogation or suspicion [45]. Institutions and teachers and faculty must address the information gap on accommodations available to the students and parents based on specific disability need, integrate inclusive teaching frameworks, and provide targeted support to ensure students, especially those with invisible disabilities, have opportunities to succeed [34].

The implications of research on institutional and systemic supports are significant. Research on effectiveness and implementation of accommodations deserves greater attention since the feasibility and sustainability of the initiatives depends on institutional commitment and available resources. Additional research is necessary to investigate the ways in which the current guidelines of halting federal oversight and funding may threaten the practice of accommodation or heighten systemic barriers for students with disabilities in U.S. schools [57].

### Limitations

Limitations of the present study include participants’ self-reported and identified disability as it relates to invisible, semi-visible, and visible disabilities. Although this study highlights important differences between participants’ diverse limitations, future research can consider operationalizing visibility of disability and gather information on specific disability groups or health conditions. Examining the role of particular invisible disabilities pertaining to neurodevelopmental, psychiatric, or chronic health conditions and unmet accommodation needs in elementary, primary, middle, or high school education will provide wider context. Moreover, while the participants in this study self-reported disability, we cannot determine how the sample compares with students who choose not to utilize disability services at all during their schooling. Our findings focused on participants’ unmet needs reflected in retrospect and do not report the frequency or extent of receipt of accommodation utilized. Additionally, the tool we used to measure needed/received academic accommodations did not include a definition of accommodations and, therefore, did not use an official definition of accommodations as referenced in relevant U.S. law such as IDEA and Section 504 of the Rehabilitation Act of 1973, limiting its applicability to these definitions. However, given the anticipated wide range of participants’ ages, we chose not to use a scale that explicitly defined accommodations based on these laws given that many participants would have attended school before they were in effect. Because we relied on participant self-report of disabilities and unmet academic accommodation needs, we cannot verify whether participants were assessed for eligibility for accommodations under IDEA or Section 504 of the Rehabilitation Act of 1973 and, therefore, whether they did not receive these accommodations due to ineligibility or because the school they attended failed to provide them even though they were eligible. Instead, we can conclude that participants who had disabilities when attending school felt they needed and did not receive specific accommodations for those disabilities. In the present study, assessing legal eligibility for individual participants would be infeasible given that we did not collect information on when participants attended school and different laws may have been in effect for participants based on when they attended school. Future studies could include more nuanced measures that capture whether participants met legal eligibility criteria for academic accommodations under the relevant laws when they attended school. Furthermore, we did not track which specific disabilities participants had and whether they had acquired additional disabilities since attending school. Future research could collect this information to more clearly articulate which specific disabilities have the most unmet academic accommodation needs.

Exploring students’ and/or parents’ motivation, contexts, barriers, and perceived helpfulness of disability accommodations utilized through mixed-methods approaches can provide valuable insights. Using online recruitment for the study might have introduced sampling bias, potentially excluding individuals with certain disabilities or underrepresenting various disability types or experiences. Indeed, given that the sample was well-educated on average with nearly half the sample having an undergraduate degree, it is possible we could have observed different results with a more diverse sample. Future work should therefore consider samples with a more diverse educational composition. Given that this study relied on retrospective reporting of childhood experiences, it is possible that participants experienced recall bias or memory distortion when reporting on past experiences. Similarly, given that this study did not collect information on when students attended school and rates of unmet academic accommodation needs may vary over time with policy and societal changes, there could have been temporal mismatches in participants’ experiences of unmet academic accommodation needs based on when they attended school. Future research could consider longitudinal designs to more accurately track unmet academic accommodation needs over time. Finally, noting disparities in graduation rates, time to graduation, post-secondary educational choices, and campus accommodations, especially for specific fields like STEM among students with disabilities [5,33], further research could consider the role of accommodations and differences in outcomes among those with visible or invisible disabilities.

## Conclusion

This study offers important implications for those assisting students with disabilities, presenting preliminary evidence into specific unmet academic accommodation needs of individuals with invisible, semi-visible, and visible disabilities. The results of the regression analysis provide evidence that disability visibility predicts unmet academic accommodation needs and attitudes towards requesting accommodations for students with disabilities, even after accounting for demographics and disability level. The probabilities observed underscore the importance of further examining invisible disability experiences of students and the other personal and social factors at play in seeking and receiving accommodations. This study also identifies opportunities for further research on initiatives, programming, and policies to enhance disabled students’ opportunities to acquire academic accommodations and foster successful educational experiences.

## Supporting information

S1 File Visbility data. (CSV)
